# Supplementary material for: A fault-intrusion-tolerant system and deadline-aware algorithm for scheduling scientific workflow in the cloud
Source: PeerJ Comput Sci. 2021 Nov 2;7:e747. doi: 10.7717/peerj-cs.747 (PMC8576554; doi:10.7717/peerj-cs.747)
Supplement: Supplemental Information 6 [file peerj-cs-07-747-s006.zip › Myproject1.1/docs/org/cloudbus/cloudsim/package-use.html]

Uses of Package org.cloudbus.cloudsim


JavaScript is disabled on your browser.


- Overview
- Package
- Class
- Use
- Tree
- Deprecated
- Index
- Help

- Prev
- Next

- Frames
- No Frames

- All Classes

# Uses of Package org.cloudbus.cloudsim

- Packages that use org.cloudbus.cloudsim

  | Package | Description |
  | --- | --- |
  |  |  |
  | --- | --- |
  | org.cloudbus.cloudsim |  |
  | org.cloudbus.cloudsim.examples |  |
  | org.cloudbus.cloudsim.examples.power |  |
  | org.cloudbus.cloudsim.examples.power.planetlab |  |
  | org.cloudbus.cloudsim.examples.power.random |  |
  | org.cloudbus.cloudsim.lists |  |
  | org.cloudbus.cloudsim.network.datacenter |  |
  | org.cloudbus.cloudsim.power |  |
  | org.cloudbus.cloudsim.power.lists |  |
  | org.cloudbus.cloudsim.provisioners |  |
  | org.cloudbus.cloudsim.util |  |
  | org.workflowsim |  |
  | org.workflowsim.clustering |  |
  | org.workflowsim.scheduler |  |
  | org.workflowsim.utils |  |
- Classes in org.cloudbus.cloudsim used by org.cloudbus.cloudsim

  Class and Description

  Cloudlet

  Cloudlet is an extension to the cloudlet.

  CloudletScheduler

  CloudletScheduler is an abstract class that represents the policy of scheduling performed by a
  virtual machine.

  CloudletSchedulerTimeShared

  CloudletSchedulerTimeShared implements a policy of scheduling performed by a virtual machine.

  Datacenter

  Datacenter class is a CloudResource whose hostList are virtualized.

  DatacenterCharacteristics

  DatacenterCharacteristics represents static properties of a resource such as resource
  architecture, Operating System (OS), management policy (time- or space-shared), cost and time
  zone at which the resource is located along resource configuration.

  File

  A class for representing a physical file in a DataCloud environment

  FileAttribute

  A class for storing related information regarding to a `gridsim.datagrid.File` entity.

  HarddriveStorage

  An implementation of a storage system.

  Host

  Host executes actions related to management of virtual machines (e.g., creation and destruction).

  HostStateHistoryEntry

  The Class HostStateHistoryEntry.

  Packet

  This class contains the structure for a network packet.

  ParameterException

  This exception is to report bad or invalid parameters given during constructor.

  Pe

  CloudSim Pe (Processing Element) class represents CPU unit, defined in terms of Millions
  Instructions Per Second (MIPS) rating.  
  **ASSUMPTION: **All PEs under the same Machine have the same MIPS rating.****

  ResCloudlet

  CloudSim ResCloudlet represents a Cloudlet submitted to CloudResource for processing.

  Storage

  An interface which defines the desired functionality of a storage system in a Data Cloud.

  UtilizationModel

  The UtilizationModel interface needs to be implemented in order to provide a fine-grained control
  over resource usage by a Cloudlet.

  Vm

  Vm represents a VM: it runs inside a Host, sharing hostList with other VMs.

  VmAllocationPolicy

  VmAllocationPolicy is an abstract class that represents the provisioning policy of hosts to
  virtual machines in a Datacentre.

  VmScheduler

  VmScheduler is an abstract class that represents the policy used by a VMM to share processing
  power among VMs running in a host.

  VmSchedulerTimeShared

  VmSchedulerTimeShared is a VMM allocation policy that allocates one or more Pe to a VM, and
  allows sharing of PEs by multiple VMs.

  VmStateHistoryEntry

  The Class VmMipsAllocationHistoryEntry.
- Classes in org.cloudbus.cloudsim used by org.cloudbus.cloudsim.examples

  Class and Description

  Cloudlet

  Cloudlet is an extension to the cloudlet.

  DatacenterBroker

  DatacentreBroker represents a broker acting on behalf of a user.

  Vm

  Vm represents a VM: it runs inside a Host, sharing hostList with other VMs.
- Classes in org.cloudbus.cloudsim used by org.cloudbus.cloudsim.examples.power

  Class and Description

  Cloudlet

  Cloudlet is an extension to the cloudlet.

  Datacenter

  Datacenter class is a CloudResource whose hostList are virtualized.

  DatacenterBroker

  DatacentreBroker represents a broker acting on behalf of a user.

  Host

  Host executes actions related to management of virtual machines (e.g., creation and destruction).

  Vm

  Vm represents a VM: it runs inside a Host, sharing hostList with other VMs.

  VmAllocationPolicy

  VmAllocationPolicy is an abstract class that represents the provisioning policy of hosts to
  virtual machines in a Datacentre.
- Classes in org.cloudbus.cloudsim used by org.cloudbus.cloudsim.examples.power.planetlab

  Class and Description

  Cloudlet

  Cloudlet is an extension to the cloudlet.
- Classes in org.cloudbus.cloudsim used by org.cloudbus.cloudsim.examples.power.random

  Class and Description

  Cloudlet

  Cloudlet is an extension to the cloudlet.
- Classes in org.cloudbus.cloudsim used by org.cloudbus.cloudsim.lists

  Class and Description

  Cloudlet

  Cloudlet is an extension to the cloudlet.

  Host

  Host executes actions related to management of virtual machines (e.g., creation and destruction).

  Pe

  CloudSim Pe (Processing Element) class represents CPU unit, defined in terms of Millions
  Instructions Per Second (MIPS) rating.  
  **ASSUMPTION: **All PEs under the same Machine have the same MIPS rating.****

  ResCloudlet

  CloudSim ResCloudlet represents a Cloudlet submitted to CloudResource for processing.

  Vm

  Vm represents a VM: it runs inside a Host, sharing hostList with other VMs.
- Classes in org.cloudbus.cloudsim used by org.cloudbus.cloudsim.network.datacenter

  Class and Description

  Cloudlet

  Cloudlet is an extension to the cloudlet.

  CloudletScheduler

  CloudletScheduler is an abstract class that represents the policy of scheduling performed by a
  virtual machine.

  Datacenter

  Datacenter class is a CloudResource whose hostList are virtualized.

  DatacenterCharacteristics

  DatacenterCharacteristics represents static properties of a resource such as resource
  architecture, Operating System (OS), management policy (time- or space-shared), cost and time
  zone at which the resource is located along resource configuration.

  Host

  Host executes actions related to management of virtual machines (e.g., creation and destruction).

  Pe

  CloudSim Pe (Processing Element) class represents CPU unit, defined in terms of Millions
  Instructions Per Second (MIPS) rating.  
  **ASSUMPTION: **All PEs under the same Machine have the same MIPS rating.****

  ResCloudlet

  CloudSim ResCloudlet represents a Cloudlet submitted to CloudResource for processing.

  Storage

  An interface which defines the desired functionality of a storage system in a Data Cloud.

  UtilizationModel

  The UtilizationModel interface needs to be implemented in order to provide a fine-grained control
  over resource usage by a Cloudlet.

  Vm

  Vm represents a VM: it runs inside a Host, sharing hostList with other VMs.

  VmAllocationPolicy

  VmAllocationPolicy is an abstract class that represents the provisioning policy of hosts to
  virtual machines in a Datacentre.

  VmScheduler

  VmScheduler is an abstract class that represents the policy used by a VMM to share processing
  power among VMs running in a host.
- Classes in org.cloudbus.cloudsim used by org.cloudbus.cloudsim.power

  Class and Description

  CloudletScheduler

  CloudletScheduler is an abstract class that represents the policy of scheduling performed by a
  virtual machine.

  Datacenter

  Datacenter class is a CloudResource whose hostList are virtualized.

  DatacenterBroker

  DatacentreBroker represents a broker acting on behalf of a user.

  DatacenterCharacteristics

  DatacenterCharacteristics represents static properties of a resource such as resource
  architecture, Operating System (OS), management policy (time- or space-shared), cost and time
  zone at which the resource is located along resource configuration.

  Host

  Host executes actions related to management of virtual machines (e.g., creation and destruction).

  HostDynamicWorkload

  The class of a host supporting dynamic workloads and performance degradation.

  Pe

  CloudSim Pe (Processing Element) class represents CPU unit, defined in terms of Millions
  Instructions Per Second (MIPS) rating.  
  **ASSUMPTION: **All PEs under the same Machine have the same MIPS rating.****

  Storage

  An interface which defines the desired functionality of a storage system in a Data Cloud.

  Vm

  Vm represents a VM: it runs inside a Host, sharing hostList with other VMs.

  VmAllocationPolicy

  VmAllocationPolicy is an abstract class that represents the provisioning policy of hosts to
  virtual machines in a Datacentre.

  VmScheduler

  VmScheduler is an abstract class that represents the policy used by a VMM to share processing
  power among VMs running in a host.
- Classes in org.cloudbus.cloudsim used by org.cloudbus.cloudsim.power.lists

  Class and Description

  Vm

  Vm represents a VM: it runs inside a Host, sharing hostList with other VMs.
- Classes in org.cloudbus.cloudsim used by org.cloudbus.cloudsim.provisioners

  Class and Description

  Vm

  Vm represents a VM: it runs inside a Host, sharing hostList with other VMs.
- Classes in org.cloudbus.cloudsim used by org.cloudbus.cloudsim.util

  Class and Description

  Cloudlet

  Cloudlet is an extension to the cloudlet.
- Classes in org.cloudbus.cloudsim used by org.workflowsim

  Class and Description

  Cloudlet

  Cloudlet is an extension to the cloudlet.

  CloudletScheduler

  CloudletScheduler is an abstract class that represents the policy of scheduling performed by a
  virtual machine.

  Datacenter

  Datacenter class is a CloudResource whose hostList are virtualized.

  DatacenterBroker

  DatacentreBroker represents a broker acting on behalf of a user.

  DatacenterCharacteristics

  DatacenterCharacteristics represents static properties of a resource such as resource
  architecture, Operating System (OS), management policy (time- or space-shared), cost and time
  zone at which the resource is located along resource configuration.

  File

  A class for representing a physical file in a DataCloud environment

  HarddriveStorage

  An implementation of a storage system.

  ParameterException

  This exception is to report bad or invalid parameters given during constructor.

  Storage

  An interface which defines the desired functionality of a storage system in a Data Cloud.

  Vm

  Vm represents a VM: it runs inside a Host, sharing hostList with other VMs.

  VmAllocationPolicy

  VmAllocationPolicy is an abstract class that represents the provisioning policy of hosts to
  virtual machines in a Datacentre.
- Classes in org.cloudbus.cloudsim used by org.workflowsim.clustering

  Class and Description

  File

  A class for representing a physical file in a DataCloud environment
- Classes in org.cloudbus.cloudsim used by org.workflowsim.scheduler

  Class and Description

  Cloudlet

  Cloudlet is an extension to the cloudlet.

  File

  A class for representing a physical file in a DataCloud environment
- Classes in org.cloudbus.cloudsim used by org.workflowsim.utils

  Class and Description

  Cloudlet

  Cloudlet is an extension to the cloudlet.

  File

  A class for representing a physical file in a DataCloud environment

- Overview
- Package
- Class
- Use
- Tree
- Deprecated
- Index
- Help

- Prev
- Next

- Frames
- No Frames

- All Classes
